# The Co-Development of Parenting Stress and Childhood Internalizing and Externalizing Problems

**DOI:** 10.1007/s10862-015-9500-3

**Published:** 2015-08-21

**Authors:** Lisanne L. Stone, Suzanne H. W. Mares, Roy Otten, Rutger C. M. E. Engels, Jan M. A. M. Janssens

**Affiliations:** Behavioural Science Institute, Radboud University Nijmegen, P.O. Box 9104, 6500 HE Nijmegen, The Netherlands

**Keywords:** Parenting stress, Internalizing problems, Externalizing problems, Transactional model

## Abstract

Although the detrimental influence of parenting stress on child problem behavior is well established, it remains unknown how these constructs affect each other over time. In accordance with a transactional model, this study investigates how the development of internalizing and externalizing problems is related to the development of parenting stress in children aged 4–9. Mothers of 1582 children participated in three one-year interval data waves. Internalizing and externalizing problems as well as parenting stress were assessed by maternal self-report. Interrelated development of parenting with internalizing and externalizing problems was examined using Latent Growth Modeling. Directionality of effects was further investigated by using cross-lagged models. Parenting stress and externalizing problems showed a decrease over time, whereas internalizing problems remained stable. Initial levels of parenting stress were related to initial levels of both internalizing and externalizing problems. Decreases in parenting stress were related to larger decreases in externalizing problems and to the (stable) course of internalizing problems. Some evidence for reciprocity was found such that externalizing problems were associated with parenting stress and vice versa over time, specifically for boys. Our findings support the transactional model in explaining psychopathology.

Adolescent internalizing and externalizing problems that persist throughout adulthood are often rooted in childhood (internalizing: Mazza et al. 2009; Costello et al. 2003; externalizing: e.g., Loeber and Hay 1997; Ashford et al. 2008; Maggs et al. 2008). Specifically, while internalizing problems in childhood have been linked to pervasive and adverse developmental outcomes, such as depression and anxiety disorders, academic underachievement, and problems with employment (Aronen and Soininen 2000; Woodward and Fergusson 2001), externalizing problems in childhood increase the risk for aggression and substance use later in life (e.g., Loeber and Hay 1997; Maggs et al. 2008). Therefore, understanding which early childhood factors are implicated in the development of in- and externalizing problems is crucial.

The development of in- or externalizing problems in childhood depends on a variety of individual and environmental factors and the interplay between these factors. As an environmental factor, parenting can be considered as *the* most important early childhood factor. From the perspective of a parent, parenting stress is one of the most prominent sources of stress, as all parents experience parenting stress to some degree (Crnic and Greenberg 1990; Hakvoort et al. 2012). As such, parenting stress is an important avenue for research. In accordance with a categorical view (i.e., yes or no; stress as the consequence of stressful life events), most current theories of parenting stress conceptualize stress as a disorder. In contrast, Daily Hassles Theory (Crnic and Greenberg 1990) posits that stress is a typical process, allowing it to fluctuate on a continuum from mild everyday stressors to very severe stress (Deater-Deckard 2004). In the present study, we do not restrict our focus on stress as a disorder, therefore we take the latter perspective.

Cross-sectional studies have shown a positive link between parenting stress and internalizing problems (Anthony et al. 2005; Costa et al. 2006; Hart and Kelley 2006; Mesman and Koot 2000; Rodriguez 2011). Increasingly, longitudinal studies confirmed these findings (Ashford et al. 2008; Bayer et al. 2006, 2008; Mäntymaa et al. 2012). However, these studies have predominantly focused on the developmental period of preschool, thereby partially neglecting the role of parenting stress and its link to internalizing problems during early childhood.

Regarding externalizing problems, the positive relation to parenting stress is well established cross-sectionally (Barry et al. 2005; Blader 2006; Creasey and Jarvis 1994; Crnic et al. 2005; Eyberg et al. 1993; Morgan et al. 2002). Data from one large study showed that child behavior of preschoolers was associated with increased levels of parenting stress (Williford et al. 2007). Further, parenting stress was positively related to externalizing problems during preschool over time (Bayer et al. 2008) and from infancy to middle childhood (Benzies et al. 2004) although these results were reported in small samples. Finally, again in a small sample, no support for such a relation was found after controlling for previous levels of externalizing problems in preschoolers (Mantymaa et al. 2012).

From these studies it remains unclear whether child behaviour affects parenting stress, and how *changes* in parenting affect problem behaviours or vice versa. Extant findings are hampered by a lack of longitudinal studies that apply a transactional perspective in which parenting stress and child problem behaviours are allowed to affect each other. Both this transactional perspective (Cicchetti 2006; Masten 2006; Sameroff 1975) and family systems theory (Minuchin 1985) propose that processes underlying developmental dysfunction are interrelated dynamically. Importantly, psychopathology is theorized to arise from complex interactions among systems between the individual and systems in which the life of the individual in embedded. Moreover, bi-directional parent and child influences have been included in theoretical models explaining psychopathology, which are referred to as parent and child effects models, respectively (e.g., Patterson 1982; Snyder and Stoolmiller 2002; see Granic and Patterson 2006). The current study adopts such a transactional perspective and examines bidirectional associations between parenting stress and internalizing and externalizing problems during early childhood (age 4–9 years).

In sum, the present study sought to answer the following research questions. First, we will examine the developmental pattern of parenting stress, internalizing and externalizing problems separately. Second, we will examine whether parenting stress is related to internalizing and externalizing problems over time. As we cannot infer directionality from this second research question, we will also examine how (i.e., in what direction) parenting stress is related to each of these problem clusters. Fourth, we will test whether there are gender differences in any of these models. The following hypotheses are tested. First, we expect no decrease or increase in internalizing problems, as these problems remain relatively stable during childhood (Maughan et al. 2008; Keiley et al. 2003), although some studies have reported an increase and decrease in internalizing problems (Costello et al. 2003; Gazelle and Ladd 2003). We expect a decrease in externalizing problems, as these problems tend to decline during childhood (Maughan et al. 2008). Parents are expected to decrease in their levels of parenting stress, as parenting stress has been found to decrease with increasing child age (Williford et al. 2007). Second, we hypothesize that parenting stress is positively related to internalizing and externalizing problems. It may be expected that children whose parents show higher levels of parenting stress show smaller decreases in their problem behavior (Deater-Deckard 2004). Also, it may be expected that parents whose children show higher levels of problem behaviors decrease to a lesser extent in their levels of parenting stress. Third, regarding directionality, we hypothesize that parenting stress predicts externalizing and internalizing problems. As pressures from below (i.e., chid behavior) have been shown to predict parenting behaviors other than parenting stress (Pomerantz and Eaton 2001), we hypothesize that child psychopathology will also predict parenting stress. Fourth, boys are expected to display more externalizing problems than girls (Dishion and Patterson 2006), while no gender differences are expected regarding internalizing problems (Ford et al. 2003).

## Method

### Sample and Procedure

Mothers of children aged 4–7 from 29 primary schools throughout the Netherlands were recruited for the Dutch “Kind in Zicht” study, in which mothers of 1339 children participated (*M*_age_ = 5.08, *SD* = 1.25, 50.1 % boys) in the first assessment. At the subsequent assessment 979 mothers participated (67 %), including 95 ‘new’ parents who did not participate in the baseline assessment for different reasons. In the third assessment wave, 819 (61 %) parents participated in the study, including 148 ‘new’ parents who did not participate in the baseline assessment. We used Mplus allowing us to estimate missing cases, leading to a final sample size of 1582. At baseline, mothers had a mean age of 36.61 (*SD =* 4.41), the majority was of Dutch origin (92.4 %) and were part of a two-parent household (89.1 %). Most mothers, 44.6 %, were highly educated with a college or university degree, 37.8 % finished vocational education, 13.7 % finished a low level of Dutch secondary school, and 4 % finished a different form of education. Attrition analyses showed that families who completed three waves (*n* = 817) did not differ from the dropouts (*n* = 522) in child age, gender, maternal educational level, family structure, internalizing, and externalizing problems, and parenting stress. Families that missed at least on of the three waves were more likely to be of non-Dutch origin (OR = 1.29, 95 % CI 1.09–1.52, *p* = .003).

We used data of three annual waves of Kind in Zicht, a large cohort study of Dutch children aged 4–7 at T1. Schools were randomly selected from the population of elementary schools in the Netherlands. Schools in the larger provinces, Noord-Holland, Zuid-Holland, Noord-Brabant and Gelderland and the four largest cities, Amsterdam, Rotterdam, The Hague and Utrecht, were oversampled. In total, 440 schools were selected. Principals of these schools first received a letter inviting them to participate in the study and subsequently were asked for participation by phone, which led to participation of 29 schools (6.6 %), containing 2558 children in two kindergarten classes, Grade 1 and 2. Schools received €1000 for participation. Teachers handed out information and consent letters to parents. Passive consent of 2360 (92.3 %) parents was obtained. Only mothers were allowed to participate in the study in order to decrease gender bias in responding. In all waves, mothers completed questionnaires either digitally or by paper and pencil.

### Measures

#### Internalizing and Externalizing Problems

The Dutch parent version of the Strengths and Difficulties Questionnaire was used at all waves to assess internalizing and externalizing problems (van Widenfelt et al. 2003). The subscale emotional symptoms (e.g., many worries, often seems worried) was used to measure internalizing problems. The conduct problems scale (e.g., often lies or cheats) was used to measure externalizing problems. Each scale contains five items and parents rated their child’s behavior on a 3-point scale ranging from 0 (not true) to 2 (certainly true). The scoring procedures used in this study are available online at www.sdqinfo.com. Higher scores indicate more problem behaviors. Cronbach’s alphas were .63, .67, .65 for emotional symptoms at T1, T2, and T3 and .48, .47, .55 for conduct problems at T1, T2, and T3. Alternative indicators of reliability based on Structural Equation Modeling – are known as Jöreskog rho or McDonalds Omega_*H.*_ Because this indicator is suggested to be more accurate when scale distributions are skewed, as is often the case in instruments measuring problem behavior, like the SDQ (Jöreskog 1971; McDonald 1978, 1999; Revelle and Zinbarg 2009; Stone et al. 2013) reliability was also calculated using omega (ω_h_). Omega values were .79, .80, .81 at T1, T2, and T3 for the emotional symptoms scale, and .71, .75, .77 at T1, T2, and T3 for the conduct problems scale. Emotional symptoms and conduct problems were positively skewed and leptokurtic. In line with Tukey’s (1977) recommendations, the least strong transformation that yielded the most symmetric distribution was chosen for each scale. For the internalizing scale the square root was taken. A logarithmic transformation was applied to the externalizing scale.

#### Parenting Stress

At all waves mothers rated the frequency of daily hassles with their child over the past 6 months (Parenting Daily Hassles: PDH; Crnic & Booth 1991; van der Wal et al. 2007). The questionnaire consists of 20 events of which the parent has to rate how often they occur (seldom, sometimes, often, constantly). A mean score was calculated with higher scores indicating higher parenting stress. Psychometric properties of the PDH have been found adequate (Crnic & Booth 1991; Rispens, Hermanns, & Meeus, 1996). Cronbach’s alphas were .77, .78, .78 at T1, T2, and T3.

#### Mental Health

Finally, we control for maternal mental health, as this is strongly related to parenting stress (Patterson 1982. Maternal mental health was related to parenting stress in the current sample (*r*s .18–.23). The degree of mental health of the mothers during the past 4 weeks was measured at the first wave with a short version of the General Health Questionnaire (GHQ; Hardy et al. 1999). Mothers rated their mental health via 12 questions (e.g., did you lose confidence in yourself? did you feel able to make decisions?) on a 4-point scale. A mean score was calculated, with higher scores indicating diminished mental health. Research into reliability and validity indicates that the GHQ has adequate psychometric properties (Ormel et al. 1991). Cronbach’s alpha was .88.

### Strategy for Analysis

Means, standard deviations and bivariate correlations of all study variables were calculated. Regarding the main analyses, first we investigated the growth of parenting stress, and internalizing and externalizing problems by employing univariate Latent Growth Curve modeling, leading to an intercept and slope for each of the three constructs (the growth parameters). Gender differences in these growth parameters were investigated by comparing a freely estimated model to a model wherein parameters were constrained to be equal for boys and girls multi-group modeling. If a significantly worse fit to the data was found for the constrained model, we employed a stepwise approach, such that each of the parameters were tested separately for gender differences by means of the *χ*^2^ difference test (http://www.statmodel.com/chidiff.html). Subsequently, we tested whether the separate growth parameters of parenting stress and internalizing problems and externalizing problems, respectively, were related to each other by combining the univariate growth models (i.e., parallel growth curves). In this step, gender differences were again tested by multi-group modeling.

To evaluate direction of effects of the associations of parenting stress with internalizing problems on the one hand, and externalizing problems on the other over time, we tested two cross-lagged path models, using Mplus version 5 (Muthén and Muthén 1998–2007). Again, gender differences were investigated by employing multi-group modeling and the *χ*^2^ difference test. All models were controlled for maternal mental health and age.

Since children from the same classes may share common behaviors (i.e., clustering), intraclass correlations (ICC) were calculated to determine the effects of class clustering. The ICC’s for internalizing problems were .019, .00, and .003 at T1, T2, and T3, respectively, .00, .047, and .016 at T1, T2, and T3 respectively, for externalizing problems, and .031, .056 and .017 at T1, T2, and T3 respectively for parenting stress. This indicates that only a small proportion of the variance could be explained by a clustering effect and therefore we decided to run the analyses without adjusting for clustering. Model fit was assessed with various fit indices, including robust chi-square with estimated degrees of freedom (df), comparative fit index (CFI; Bentler 1990), root mean squared error of approximation (RMSEA; Byrne 1998), and Tucker–Lewis index (TLI; Tucker and Lewis 1973). Several models we tested were saturated (*χ*^2^ (0) = 0.00), therefore we did not report fit indices for these models. Although this procedure is employed frequently, we acknowledge there is debate on the question whether saturated models should be interpreted. We follow Muthén’s recommendation that significance of pathways within the model can be interpreted without fit indices (see for a discussion: http://www.statmodel.com/discussion/messages/11/2127.html?1397836729).

## Results

### Descriptive Statistics

Internalizing and externalizing problems were moderately related to parenting stress across time (Table 1). Internalizing and externalizing problems but also parenting stress were strongly correlated across time, indicating high stability. Mental health was correlated positively over time and weakly associated with internalizing and externalizing problems, and moderately with parenting stress. Age was positively correlated to internalizing problems at T1 and T2, indicating that for older children more internalizing problems were reported. However, age correlated negatively to parenting stress, such that less parenting stress was reported for older children. At T1, a small correlation was found between externalizing problems and age. For older children, less externalizing problems were reported. Regarding gender, more externalizing problems were reported for boys.

**Table 1:** Correlations between all study variables (*N* = 1582)

		*M (SD)*	1	2	3	4	5	6	7	8	9	10	11
1	Internalizing T1	1.59 (1.81)	–										
2	Internalizing T2	1.68 (1.87)	.53**	–									
3	Internalizing T2	1.67 (1.85)	.49**	.54**	–								
4	Externalizing T1	1.28 (1.44)	.19**	.14**	.18**	–							
5	Externalizing T2	1.14 (1.35)	.13**	.25**	.26**	.54**	–						
6	Externalizing T3	1.02 (1.36)	.15**	.22**	.32**	.46**	.55**	–					
7	Parenting Stress T1	1.49 (.26)	.22**	.21**	.23**	.34**	.34**	.29**	–				
8	Parenting Stress T2	1.49 (.27)	.17**	.31**	.28**	.31**	.36**	.32**	.65**	–			
9	Parenting Stress T3	1.45 (.25)	.17**	.26**	.31**	.32**	.35**	.39**	.59**	.67**	–		
10	Mental Health T1	1.44 (2.53)	.10**	.19**	.12**	.14**	.09**	.14**	.20**	.23**	.18**	–	
11	Age	5.08 (1.25)	.11**	.12**	.04	−.06*	−.01	.01	−.11**	−.09*	−.14**	−.03	–
12	Gender	–	.04	.02	−.03	−.05*	−.15**	−.15**	−.01	−.02	−.03	−.00	−.02

Gender is coded as 0 = boys, 1 = girls. ** p < .01, * *p* < .05

### Basic Growth Curves

Growth parameter estimates for the basic growth curve models are reported in Table 2. Fit statistics for the model investigating the shape and growth of internalizing problems were satisfactory (*χ*^2^(3) = 10.76, *p* = 0.013; *CFI* = 0.987; *RMSEA* = 0.041 (CI = 0.017–0.069); TLI = 0.957). No gender differences were found regarding initial level (intercept), change over time (slope), the variance around the initial level and change over time, or covariance among initial level and change over time, indicating that boys and girls do not differ in their development of internalizing problems (Δ*χ*^2^(5) = 4.50, *p* > .90). Internalizing problems remain stable over time, which was indicated by a non-significant slope. Significant inter-individual differences in initial level, but not in change of these problems were found, suggesting that children differ in their level of internalizing problems at baseline. The initial level of, and change in internalizing problems were not related, suggesting that the level of internalizing problems is not associated with change in these problems.

**Table 2:** Unstandardized growth parameters of the basis growth curves

	Mean				Variance				Covariance
	Intercept	*T* (S.E.)	Slope	*T* (S.E.)	Intercept	*T* (S.E.)	Slope	*T* (S.E.)	Intercept-Slope
Internalizing Problems	.971**	45.28 (.02)	.018	1.27 (.01)	.386**	10.27 (.04)	.028	1.48 (.02)	−.025
Externalizing Problems	.292**/.26**	**30.05 (.01)/** *27.40 (.00)*	−.014*/−.039**	**−2.10 (.00)**/−*6.53 (.00)*	.04**	10.74 (.00)	.005**	2.84 (.00)	−.005*
Parenting Stress	1.50**	210.36 (.01)	−.016**	−3.97 (.00)	.052**	10.79 (.00)	.005*	2.59 (.00)	−.005*

Coefficients in bold represent parameters for boys, coefficients in italics represent parameters for girls, which are statistically

different.** p < .01, * *p* < .05

Regarding externalizing problems, again an adequate fit to the data was found (*χ*^2^(3) = 3.92, *p* = 0.27; *CFI* = 0.998; *RMSEA* = 0.014 (CI = 0.00–0.048); TLI = 0.994). Several gender differences were found regarding initial level and change in externalizing problems over time. Boys showed higher levels of externalizing problems at baseline than girls (Δ*χ*^2^(1) = 5.43, *p* < .025), and girls showed a steeper decrease in externalizing problems than boys (Δ*χ*^2^(1) = 8.09, *p* < .005). No gender differences were found regarding the variance around the initial level and change over time, or covariance among initial level and change over time (Δ*χ*^2^(3) = 4.86, *p* > .90). Significant inter-individual differences in initial level, and in change of these problems were found, suggesting that children differ in both their level of externalizing problems at baseline and the decrease in these problems. The initial level of, and change in externalizing problems were negatively related, such that children with higher levels of externalizing problems at baseline showed smaller decreases of externalizing problems over time.

Regarding parenting stress, a satisfactory fit to the data was found (*χ*^2^(3) = 18.68, *p* = 0.000; *CFI* = 0.978; *RMSEA* = 0.059 (CI = 0.035–0.086); TLI = 0.928). No gender differences were found regarding initial level, change over time, the variance around the initial level and change over time, or covariance among initial level and change over time (Δχ^2^(5) = 2.98, *p* > .90). Significant inter-individual differences in initial level, and in change of parenting stress were found, suggesting that mothers differ in both their level of parenting stress at baseline and the decrease in the parenting stress. The initial level of, and change in parenting stress were negatively related, such that mothers with higher levels of parenting stress at baseline showed smaller decreases of parenting stress over time.

### Parallel Growth Curves

Correlations between growth parameter estimates of parenting stress and internalizing problems, and externalizing problems respectively are reported in Table 3. A satisfactory fit was found for the model investigating the interrelation of parenting stress and internalizing problems (*χ*^2^(11) = 50.53, *p* = 0.000; *CFI* = 0.974; *RMSEA* = 0.049 (CI = 0.036–0.063); TLI = 0.933). No gender differences were found regarding interrelations between internalizing problems and parenting stress (Δχ^2^(3) = 1.54, *p* > .10). Higher levels of baseline levels of internalizing problems were associated with higher levels of parenting stress and the course (i.e. stability) of internalizing problems was related to stronger decreases in parenting stress. The level of internalizing problems was not related to the decrease of parenting stress. Also, the level of parenting stress was not related to the course (i.e. stability) of internalizing problems.

**Table 3:** Correlations between the growth parameters in the parallel growth curves

		Parenting Stress	
		Intercept	Slope
Internalizing Problems	Intercept	.049**	−.001
	Slope	.003	.006**
Externalizing Problems	Intercept	.022**	−.001
	Slope	−.002	.002**

** p < .01, * *p* < .05. Coefficients in parentheses represent the unstandardized correlations

Fit statistics for the model investigating the interrelation of parenting stress and externalizing problems were satisfactory (*χ*^2^(11) = 35.34, *p* = 0.000; *CFI* = 0.984; *RMSEA* = 0.038 (CI = 0.025–0.053); TLI = 0.961). Higher baseline levels of externalizing problems were associated with higher baseline levels of parenting stress and decreases in externalizing problems were related to larger decreases in parenting stress. The level of externalizing problems was not related to the decrease of parenting stress; also, the level of parenting stress was not related to the decrease of externalizing problems. No gender differences were found regarding interrelations between externalizing problems and parenting stress (Δ*χ*^2^(3) = 6.210, *p* > .10).

### Cross-Lagged Models

The model in which the direction of effects of parenting stress on internalizing problems and vice versa was tested was saturated. Internalizing problems showed moderate to strong stability throughout childhood (*r* .27–.51, *p* < .000), as did parenting stress (*r* .30–.66, *p* < .000). Internalizing problems and parenting stress were positively concurrently related at each time point, indicating that more internalizing problems are related to more parenting stress. Cross-lagged paths from internalizing problems to parenting stress were all non-significant, indicating that there were no child effects of internalizing problems on parenting stress. Parenting stress at T1 was related to internalizing problems at T2, and a statistical trend was found for the cross-lagged path of parenting stress at T1 to internalizing problems at T3, such that more parenting stress was related to more subsequent internalizing problems. Parenting stress at T2 was not related to internalizing problems at T3. No gender differences were found regarding cross-lagged paths (Δ*χ*^2^(6) = 4.735, *p* > .10).

The model in which the interrelations between parenting stress and externalizing problems were tested was also saturated. Externalizing problems showed moderate stability throughout childhood (*r* .21–.48, *p* < .000). Externalizing problems and parenting stress were positively concurrently related at each time point, indicating that more externalizing problems are related to more parenting stress. The cross-lagged paths differed across gender (Δ*χ*^2^(6) = 12.865, *p* < .05). For boys, significant child-on-parent effects were found. Externalizing problems at T1 were related to parenting stress at T2 and externalizing problems at T2 were related to parenting stress at T3, such that more externalizing problems were related to more subsequent parenting stress. Also, parent-on-child effects were detected, such that parenting stress at T1 was related to externalizing problems at T2, and parenting stress at T2 was related to externalizing problems at T3. Thus, more parenting stress was related to more subsequent externalizing problems. For girls, externalizing problems at T1 were related to parenting stress at T3, such that more externalizing problems were related to more subsequent parenting stress. This relation was not found at the other time points. Parenting stress at T1 was related to externalizing problems at T2, such that more parenting stress was related to more subsequent externalizing problems. Again, this relation was not found at other time points.

## Discussion

The present study examined the developmental patterns of parenting stress, internalizing and externalizing problems and the relations between parenting stress and internalizing and externalizing problems over time in children aged 4–9 from a large community sample. Additionally, we examined how parenting stress was related to each of these problem clusters. These associations were compared between boys and girls and we controlled for maternal mental health.

### Development of Problem Behaviors

It is well known that externalizing problems tend to decrease during childhood (Maughan et al. 2008). These findings are replicated and extended by the current study, by differentiating the growth rates for boys and girls and by investigating inter-individual variability. In line with the literature, boys show higher levels of externalizing problems at age 4–7 and decrease slower than girls (Dishion and Patterson 2006). Regarding internalizing problems, no such gender differences were found (Ford et al. 2003). Furthermore, this study showed that there are inter-individual differences in the initial level of externalizing and internalizing problems.

Regarding developmental course, children varied in their decrease rate regarding externalizing, but not regarding internalizing problems. This is interesting, as distinct trajectories in the development of internalizing problems have been reported previously (Sterba et al. 2007; Côté et al. 2009), indicating differences in the development of internalizing problems. However, several differences in study design and sample may account for these diverging results. Côté et al. focused on the preschool period and Sterba et al. focused on children aged 2–11. Possibly, differences in the course of internalizing problems are pushed by early developmental changes in internalizing problems, and not so much by changes during middle childhood. This statement is speculative though and requires empirical testing. Further, more comprehensive instruments for assessing internalizing problems were utilized in these studies, Côté et al. used the Children’s Depressive and Anxiety Symptoms in preschool (DAS) and the Child Behavior Check List (CBCL) was used by Sterba et al., whereas the SDQ that we used was designed as a screening instrument, and hence, shorter. This may have led to more sensitive measurement of internalizing problems, and thus of more variation in reports of internalizing problems. If anything, our results call for the need for further validation of the course of internalizing problems. Regarding associations of the initial level of problems and its course, children with higher levels of externalizing problems at age 4–7 showed smaller decreases of externalizing problems over time. These results confirm the theory of antisocial development that more severe externalizing problems are associated with a worse outcome (Loeber 1991). For internalizing problems, initial level of these problems was not related to its course.

### Development of Parenting Stress and its Relation to Problem Behaviors

Only one study investigated the developmental course of parenting stress (Williford et al. 2007). Our results concur with this study in the sense that parenting stress decreased over time and inter-individual differences in the initial level and in the course of parenting stress were found. These results mesh with extant literature showing that children’s independence increases in middle childhood, thereby decreasing the strain and demands on parents (Berk 2012). Also, the initial level of parenting stress was negatively related to its course, implying that higher reports of parenting stress at baseline were related to smaller decreases in parenting stress over time. This may be explained by the role of cognitions about parenting stress (Lazarus 1999), wherein these dysfunctional cognitions maintain perceptions of stress (Deater-Deckard et al. 2005). Thus, when parenting stress is present at some point, it is likely that dysfunctional cognitions maintain perceptions of stress, thereby reducing the decrease in perceived stress.

The current study expanded on Williford et al. (2007), by investigating how the development of parenting stress was related to both internalizing and externalizing problems. As expected, parenting stress and internalizing and externalizing problems were interrelated at baseline (e.g., Crnic et al. 2005; Rodriguez 2011). The decrease in parenting stress was not affected by the initial level of internalizing and externalizing problems, which was in contrast with our hypothesis that higher levels of parenting stress are associated with smaller decreases in problem behavior (Deater-Deckard 2004). These findings suggest that other factors influence the course of parenting stress. Possibly, socioeconomic stressors, such as economic hardship, affect aspects of personality, by increasing ineffective coping styles, (Crnic et al. 2005; Warfield 2005), which in turn affect the course of parenting stress. However, the course of parenting stress was related to the course of internalizing and externalizing problems, suggesting that these constructs co-evolve during childhood. Together, these findings suggest a complex interplay between factors related to the child and to the parent, which is in accordance with a transactional model (Sameroff 1975; Cicchetti 2006; Minuchin 1985).

As for directionality, consistent patterns in parenting stress and externalizing problems were found for boys above and beyond stability of these problems and while taking into account concurrent relations. For girls, the pattern was less consistent but still in accordance with a bidirectional model, as parenting stress at baseline impacted externalizing problems 1 year later, and externalizing problems at baseline impacted parenting stress 2 years later. These results fit well with the theoretical notion that both parents and children affect each other’s development, as described in the bidirectional model (Sameroff 1975; Cicchetti 2006; Minuchin 1985). However, regarding internalizing problems, no support was found for the child effects model, as internalizing problems did not affect subsequent parenting stress above and beyond stability of these problems and while taking concurrent relations into account. Some support was found for a parent effects model, as parenting stress at baseline was associated with internalizing problems 1 year later. These results diverge from those found in the growth models, and may be explained by analytic principles. While growth models take inter-individual differences into account, in cross-lagged models only the group level is included. Moreover, time is used in a different way in these analyses, as in cross-lagged analyses each time point is used separately, whereas in growth models change is modeled as change across time.

### Limitations and Future Directions

First, given the consistent empirical finding that informants tend to disagree regarding problem behavior and parenting (De Los Reyes and Kazdin 2005; Taber 2010), the results of this study may be informant specific, that is specific to mothers. As such, our results should not be generalized to fathers and to other informants such as children. Also, the possibility that our results reflect shared method variance cannot be ruled out (De Los Reyes and Kazdin 2005). Future studies should include reports of multiple informants. Interestingly, such a framework may actually help to explain how maladjustment develops (De Los Reyes 2012), instead of making the results harder to interpret. For example, parent–child discrepancies regarding parenting stress may represent features of the parent–child relationship, which in turn affect child outcomes (Goodman et al. 2010). Moreover, analytical techniques enable partitioning out non-shared variance among informant’s reports, and testing whether these reflect unique information, instead of treating this variance as error (e.g., Bartels et al. 2007). Relatedly, given the large sample size, only questionnaires were used in this study. Combining questionnaire data with observations would provide a more thorough and valid test of interrelations between parenting stress and problematic behaviors. Second, as stated above, our sample is normative and relatively low risk. This means that our results should not be generalized to high risk or clinical samples per se. However, the spectrum hypothesis states that differences between normative and clinical samples primarily lie in mean level differences and in strength of associations, but not in structural or qualitative differences (Costa and Widiger 2002; Van Leeuwen et al. 2007). By including both normative and clinical samples in future studies, this hypothesis can be tested regarding relationships between parenting stress and problem behaviors.

## Conclusions

**Fig. 1:**
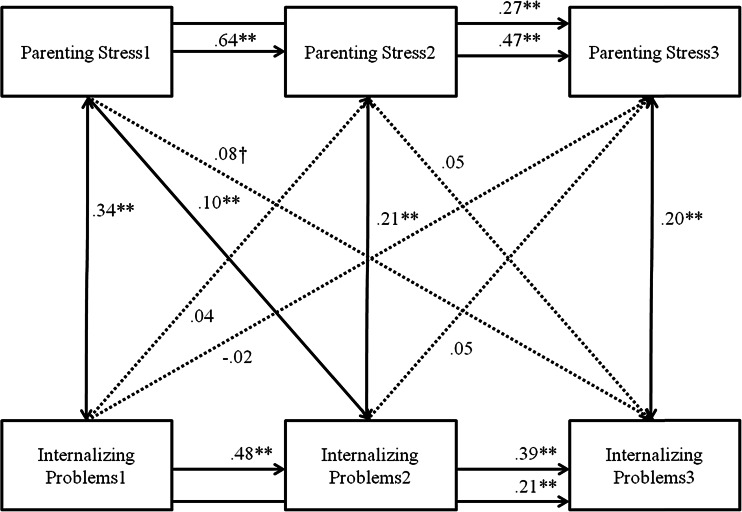
Cross-lagged Model between Internalizing Problems and Parenting Stress. *Note.* Numbers after variable names refer to data waves. ** p < .01, * *p* < .05, † *p* < .10

**Fig. 2:**
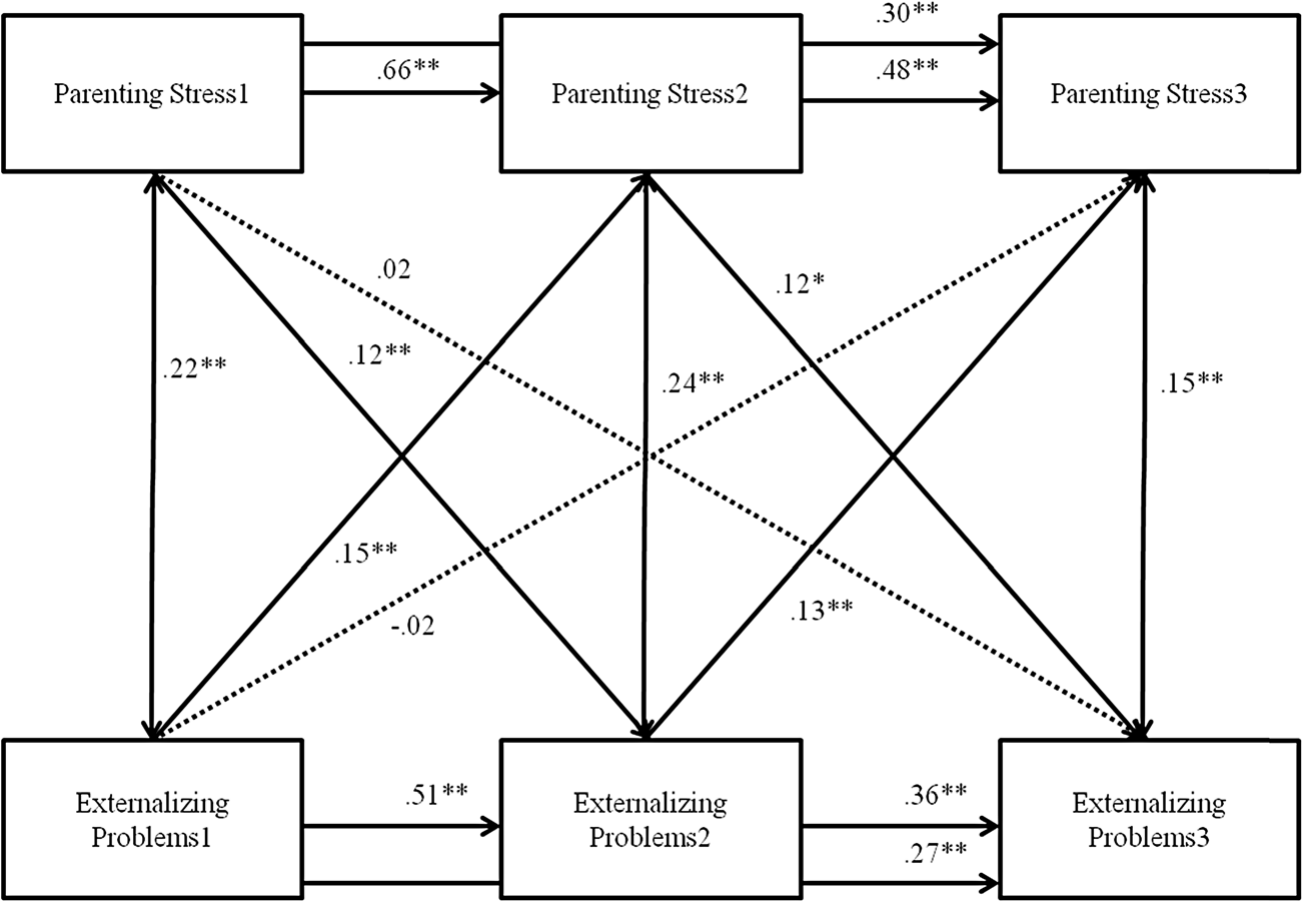
Cross-lagged Model between Externalizing Problems and Parenting Stress, for boys. *Note.* Numbers after variable names refer to data waves. ** p < .01, * *p* < .05

**Fig. 3:**
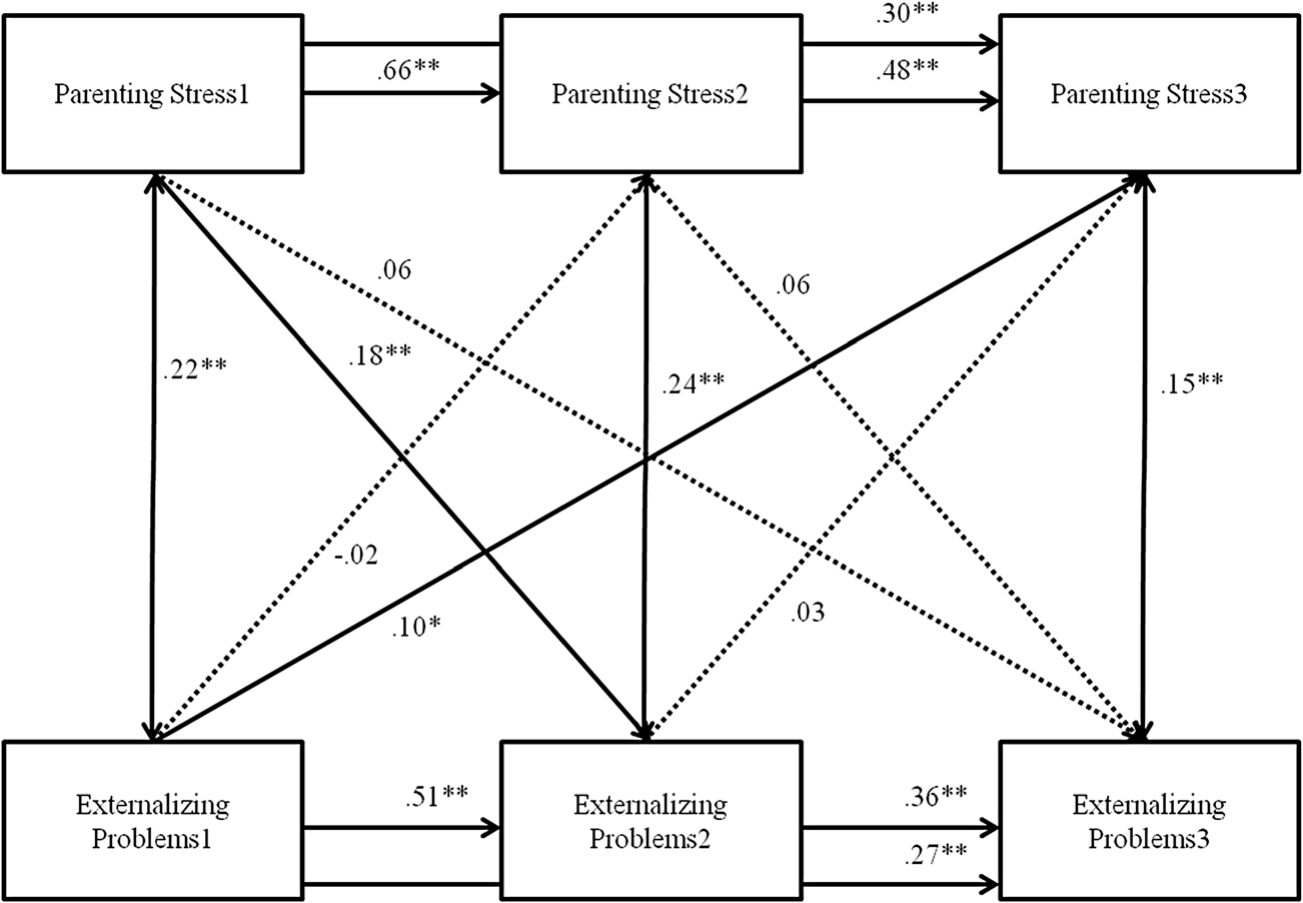
Cross-lagged Model between Externalizing Problems and Parenting Stress, for girls. *Note.* Numbers after variable names refer to data waves. ** p < .01, * *p* < .05

This study employed a developmental perspective to investigate the interrelatedness between parenting stress and internalizing and externalizing problems. Given the limited studies investigating these interrelations dynamically, the current study is an important contribution to the extant literature. We showed that parenting stress is not a static construct and decreases during early childhood, similarly for boys and girls. In addition, we showed that internalizing problems remain stable in early childhood, and replicated findings regarding decreasing externalizing problems. Finally, parenting stress and internalizing and externalizing co-evolve, thereby mutually influencing each other. Regarding internalizing problems, however, child effects were not found. This finding calls for the need for future studies to further disentangle the interrelatedness of parenting and internalizing problems (Figs. 1, 2 and 3).
